# Tail-anchored membrane protein SLMAP3 is essential for targeting centrosomal proteins to the nuclear envelope in skeletal myogenesis

**DOI:** 10.1098/rsob.240094

**Published:** 2024-10-09

**Authors:** Ana Paula Dias, Taha Rehmani, Maysoon Salih, Balwant Tuana

**Affiliations:** ^1^ Department of Cellular and Molecular Medicine, University of Ottawa, Ottawa, Canada K1H 8M5

**Keywords:** SLMAP3, skeletal myogenesis, non-centrosomal microtubule-organizing centre, Golgi, LINC complex, nuclear envelope

## Abstract

The positioning and communication between the nucleus and centrosomes are essential in cell division, differentiation and tissue formation. During skeletal myogenesis, the nuclei become evenly spaced with the switch of the microtubule-organizing centre (MTOC) from the centrosome to the nuclear envelope (NE). We report that the tail-anchored sarcolemmal membrane associated protein 3 (SLMAP3), a component of the MTOC and NE, is crucial for myogenesis because its deletion in mice leads to a reduction in the NE-MTOC formation, mislocalization of the nuclei, dysregulation of the myogenic programme and abnormal embryonic myofibres. SLMAP3^−/−^ myoblasts also displayed a similar disorganized distribution of nuclei with an aberrant NE-MTOC and defective myofibre formation and differentiation programming. We identified novel interactors of SLMAP3, including pericentrin, PCM1 (pericentriolar material 1), AKAP9 (A-kinase anchoring protein 9), kinesin-1 members Kif5B (kinesin family member 5B), KCL1 (kinesin light chain 1), KLC2 (kinesin light chain 2) and nuclear lamins, and observed that the distribution of centrosomal proteins at the NE together with Nesprin-1 was significantly altered by the loss of SLMAP3 in differentiating myoblasts. SLMAP3 is believed to negatively regulate Hippo signalling, but its loss was without impact on this pathway in developing muscle. These results reveal that SLMAP3 is essential for skeletal myogenesis through unique mechanisms involving the positioning of nuclei, NE-MTOC dynamics and gene programming.

## 1. Introduction

Mechanisms that guide the positioning of the nucleus are critical for cell migration, division and tissue formation. The linker of nucleoskeleton and cytoskeleton (LINC) complex comprising klarsicht/ANC-1/Syne-1 homology (KASH) and Sad1/UNC-84 (SUN) proteins are believed to bridge the nuclear membrane to transmit mechanical information from the cytoskeleton to influence nuclear positioning as well as impact the nuclear lamina and chromatin organization [1,2]. Skeletal muscle formation is a fascinating example of the radical cellular cytoskeleton reorganizations that cells can undergo, where the proliferative progenitor myoblast cells differentiate and fuse, forming a syncytium that is called myotube [3]. In this process, a cytoskeletal remodelling takes place before myoblast fusion [4–6], where microtubule-organizing centre (MTOC) proteins from the centrosome relocate to the nuclear envelope (NE) [4,7–9], in a process dependent on the LINC complex member Nesprin-1 [10]. It is hypothesized that this MTOC switch is important for the proper localization of nuclei in developing myotubes [11,12]. Nuclear mispositioning in myofibres has been associated with muscular dysfunctions, including Emery–Dreifuss muscular dystrophy (EDMD) and centronuclear myopathy (CNM) [11,12], which are characterized by muscle weakness and wasting [13,14]. Following the MTOC switch in differentiating myoblast, the Golgi apparatus relocalizes in perinuclear regions (PEs), forming a Golgi belt-like ring structure [4,5,8,15], and itself also becomes an MTOC [16–18]. The endoplasmic reticulum (ER) exit sites (ERES) are remodelled together with the Golgi [5,15,19]. The remodelling of Golgi and ERES suggests that the secretory pathway surrounding the nucleus must be also important for the skeletal muscle function, such as the proper transport of M-cadherin to cellular membrane and contribute to myoblast fusion [19]. Centrosomal proteins including pericentrin, PCM1 and AKAP9 have been reported to be important for the centrosome-NE MTOC switch and Golgi remodelling [10,18,20,21].

Previously, we defined the sarcolemmal membrane associated protein 3 (SLMAP3) as a component of the centrosome, where it localizes via its N-terminal sequences encompassing the forkhead-associated domain (FHA) [22]. SLMAP3 can also target the perinuclear membrane via its hydrophobic C-terminal tail anchor [23,24]. SLMAP3 has also been found in the striatin-interacting phosphatase and kinase (STRIPAK) complex [25,26] and linked to the Hippo signalling by inhibiting MST kinase, and consequently activating Yes1 associated transcriptional regulator (YAP) and transcriptional coactivator with PDZ-binding motif (TAZ) to induce proliferation [27–30]. To elucidate the role of SLMAP3 *in vivo*, we devised a strategy to specifically delete the SLMAP3 isoform in embryonic and post-natal heart muscle, but these mice exhibited normal physiology most likely due to the presence of the other SLMAP isoforms in cardiomyocytes [31,32]. Splicing mechanisms generate many SLMAP isoforms that are expressed in a developmental and tissue-specific manner [33,34]. Here we report the generation of global SLMAP3 knockout^(−/−)^ mice, which displayed stunted growth characteristics with widespread organ deficits leading to lethality. SLMAP3^−/−^ embryos presented abnormal skeletal muscle and molecular analysis indicated downregulation of genes involved in skeletal muscle development. Furthermore, the deletion of the SLMAP gene in mouse myoblasts, which express only SLMAP3, caused aberrant differentiation and myotube formation. A major impact on the distribution and positioning of the nuclei and a defective centrosome-NE MTOC switch was evident *in vivo*, and cultured myotubes devoid of SLMAP3. The loss of SLMAP3 also led to a reduction in the Golgi apparatus remodelling to PEs in myoblasts. We identified SLMAP3 in a novel complex with components of the MTOC, kinesin-1 motor proteins and nuclear lamins. AKAP9, pericentrin and PCM1, as well as Kinesin-1 members Kif5B, KLC1 and KLC2, are known to contribute to the proper positioning of nuclei in developing myotubes [20,35–37]. The ability of SLMAP3 to localize and assemble with components from the various organelles implies that it can influence their functions and organization in cell biology. The impact on genes associated with muscle development and new interactions of SLMAP3 reveal an essential role for this protein through mechanisms linking centrosomal and nuclear organization and activity.

## 2. Results

### 2.1. SLMAP3 is present in the centrosome and perinuclear membrane of skeletal myoblasts

The *slmap* gene encodes multiple protein isoforms, which display C-terminus transmembrane domain (TM) and coiled-coil (CC) domains along their structure [22,33,34] (electronic supplementary material, figure S1*a*). The TM domain anchors SLMAP to ER, mitochondria and perinuclear membranes [23,23,24], whereas the CC domains are responsible for protein–protein interaction, including its own self-assembly [24,38,39]. The SLMAP3 isoform additionally has an N-terminus containing an FHA (electronic supplementary material, figure S1*a*), which is necessary to target the centrosome [22,40]. SLMAP3 is ubiquitously expressed whereas the other isoforms are expressed in a tissue-specific manner, with muscle subtypes exhibiting diverse isoforms [33,34]. SLMAP3 localization in skeletal myoblasts was examined by transducing C2C12 cells with adenovirus carrying SLMAP3 tagged with green fluorescent protein (GFP-SLMAP3) and inducing their differentiation in low serum medium for 3 days (electronic supplementary material, figure S1*b*). In undifferentiated and differentiated myoblasts (stained for MyoG), GFP-SLMAP3 localized in the centrosome (arrows) and NE (arrowhead). Upon differentiation, GFP-SLMAP3 localization in the NE becomes more evident and correlates with pericentrin localization at this structure (electronic supplementary material, figure S1*b*). The cytoplasmic localization of GFP-SLMAP3 corresponds to the ER, which was stained for calnexin, with Pearson’s *r* = 0.705 and Mander’s coefficients *M*1 = 0.991 and *M*2 = 0.963 (electronic supplementary material, figure S1*c*). The Golgi staining with anti-GM130 showed poor colocalization of GFP-SLMAP3 with this organelle (Pearson’s *r* = 0.241). These data demonstrate that in skeletal myoblasts SLMAP3 localizes in the centrosome, NE and in the ER, but not in the Golgi apparatus.

### 2.2. Genes downregulated in SLMAP3^−/−^ mice embryos enrich for muscle developmental processes

**Figure 1.**
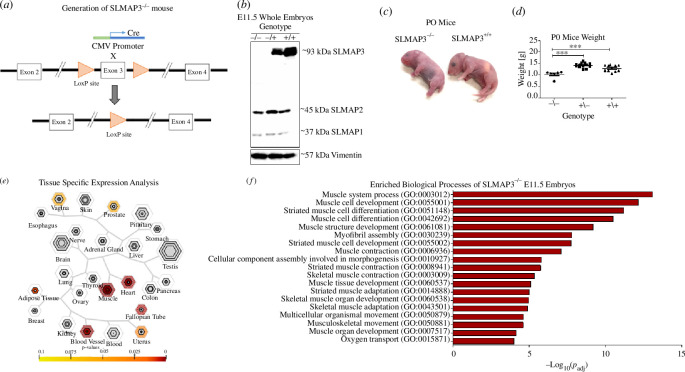
Genes downregulated in SLMAP3^−/−^ mouse embryos enrich for muscle developmental processes. (*a*) Mice carrying floxed *slmap* gene [31] were bred with mice carrying cre under CMV promoter to nullify SLMAP3 expression. (*b*) Western blot with an anti-SLMAP antibody that detects all isoforms showing SLMAP expression in SLMAP3^−/−^, SLMAP3^−/+^ and SLMAP3^+/+^ E11.5 embryos. (*c*) Phenotype of SLMAP3^−/−^ P0 mice, with short limbs and tails. (*d*) SLMAP3^−/−^ pups have significantly reduced body weight when compared to SLMAP3^+/−^ or SLMAP3^+/+^ animals. (*e*) The tissue-specific expression analysis (TSEA) shows a significant enrichment of muscle tissues of the downregulated genes in SLMAP3^−/−^ embryos in all specificity index threshold (pSI) stringencies. (*f*) The downregulated genes in RNA-seq from SLMAP3^−/−^ embryos enrich for muscle developmental processes. Three SLMAP3^−/−^ and four wild-type (WT) embryos were sequenced. Gene ontology enrichment performed using g:Profiler.

Since the *in vivo* function of SLMAP remains elusive, we have generated a floxed mouse line for global ablation of SLMAP3 using the Cre-Lox system. The loxP sites were inserted flanking exon 3 of the SLMAP gene (reference ENSMUST00000139075.8; figure 1*a*) as described [31], and these mice were crossed with CMV-Cre mice to create the SLMAP3^−/−^ animals (figure 1*b*). The resulting mice exhibit late embryonic/perinatal lethality due to underdeveloped essential organs, such as lungs, and display other phenotypes including short tail and limbs (figure 1*c*) and reduced body weight (figure 1*d*).

For a better characterization of SLMAP3^−/−^ embryonic phenotype, we performed RNA sequencing of whole E11.5 embryos from SLMAP3^−/−^ and wild-type (WT) animals (electronic supplementary material, table S1). The significant genes with log_2_ fold change below or equal to −0.75 were selected as query for the tissue-specific expression analysis (TSEA) tool [41,42] and for gene ontology enrichment analysis on g:Profiler [43]. TSEA results indicate the strongest enrichment of muscle tissues, with statistical significance reached in all specificity index thresholds (pSI; figure 1*e*), and the topmost significant enriched biological processes detected on g:Profiler are associated with muscle development (figure 1*f*). The data are also available in the Gene Expression Omnibus (GEO) genomics data repository with the accession number GSE230748.

### 2.3. SLMAP3 exists in association with MTOC proteins and nuclear lamins

**Figure 2.**
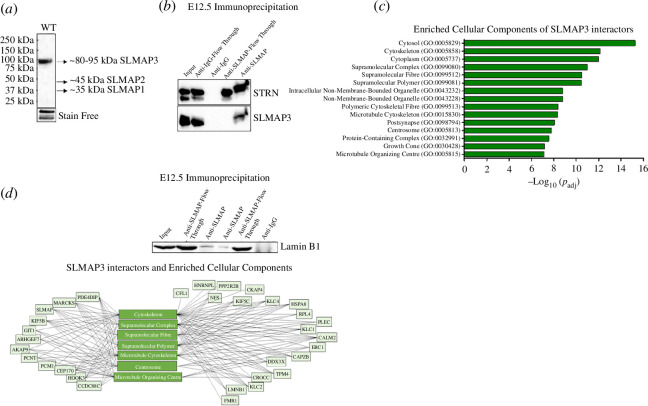
SLMAP3 interacts with MTOC proteins. (*a*) SLMAP3 (80–95 kDa) is the only isoform present in E12.5 mouse brains, as indicated by the western blot analysis and the smaller isoforms SLMAP1 (35 kDa) and SLMAP2 (45 kDa) are undetected. (*b*) Validation of IP–MS, confirming the interaction of SLMAP3 with Lamin B1 and STRN. (*c*) Cellular component enrichment of SLMAP3 interactors, identified by IP–MS, performed on g:Profiler. (*d*) SLMAP3 interactors enrich for cytoskeleton, supramolecular complex, supramolecular fibre, supramolecular polymer, microtubule cytoskeleton, centrosome and MTOC cellular component.

To investigate SLMAP3 interactors *in vivo*, we performed immunoprecipitation followed by mass spectrometry (IP–MS) with protein lysates from E12.5 mouse brain, an organ where only the SLMAP3 isoform is expressed (figure 2*a*). We identified SLMAP3 in a new complex with AKAP9, PCNT, PCM1, Kinesin-1 subunits (Kif5B, KLC1 and KLC2) and lamin B as well as the expected STRIPAK component striatin (STRN) (electronic supplementary material, table S2). The IP–MS data were validated by IP with anti-SLMAP and western blot with anti-STRN and anti-lamin B1, both of which were detected in the IP–MS (figure 2*b*). The SLMAP3 interactors were used for gene ontology enrichment analysis on g:Profiler. The top cellular components enriched include cytoskeleton (GO:0005856) and supramolecular complex (GO:0099080), but also centrosome (GO:0005813), microtubule cytoskeleton (GO:0015630) and MTOC (GO:0005815; figure 2*c*). The proteins enriching these cellular components are shown in figure 2*d*. These proteomics data are available via ProteomeXchange with identifier PXD041687, and the exclusive unique spectrum counts of the interactors are given in electronic supplementary material, table S2.

It is notable that SMAP3 is a component of the MTOC and localizes to the NE and its newly identified interactors (PCNT, PCM1, AKAP9, kinesin-1 and lamin B) also reside at these locations, providing credence to the IP–MS data. These proteins play important roles during skeletal muscle development, by participating in nuclear positioning and in centrosomal MTOC switch to the NE [11,12]. Considering the downregulation of genes associated with muscle development and the identification of the aforementioned proteins in complex with SLMAP3, we speculated that depletion of SLMAP3 impairs muscle development by affecting the function of MTOC.

### 2.4. SLMAP3^−/−^ myoblasts display differentiation and myotube formation defects

To elucidate the role of SLMAP3 in skeletal muscle development, we generated SLMAP3^−/−^ C2C12 myoblasts with CRISPR/Cas9, as these cells represent an excellent model of myogenesis. Two guide RNAs were designed to target exon 3 of SLMAP (reference ENSMUST00000139075.8) next to the predicted start codon (electronic supplementary material, figure S2*a*), keeping the same strategy of exon 3 disruption as in our Cre-Lox SLMAP3^−/−^ mouse. A monoclonal colony of each guide was selected for further experiments, designated KO1 and KO2 (colony 90 and 14, respectively), with depletion of the SLMAP3 protein assayed by western blots (electronic supplementary material, figure S2*b*) and immunofluorescence staining of SLMAP3 in KO2 (electronic supplementary material, figure S2*c*).

**Figure 3.**
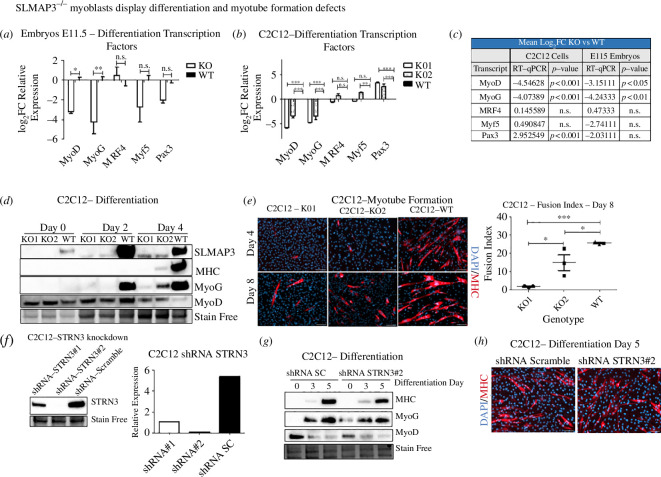
SLMAP3^−/−^ myoblasts display differentiation and myotube formation defects. (*a*) RT-qPCR shows that KO1 and KO2 cells have altered the expression of MRFs. (*b*) The RNA-seq from E11.5 was validated by RT-qPCR, where we confirmed the reduced expression of myod1 and myog. (*c*) Comparison of the mean log_2_ fold change (log_2_FC) values from the transcripts of both KO C2C12 cells and E11.5 embryos obtained by RT-qPCR. The C2C12 cells and embryos null for SLMAP3 have significantly reduced myod and myog expression. (*d*) Differentiation assay was performed with low serum media, and protein lysates were collected on days 0, 2 and 4. The western blot shows that C2C12 myoblasts lacking SLMAP3 have reduced MHC and MyoG protein levels. (*e*) Myotube formation in SLMAP^−/−^ C2C12 cells is impaired. On differentiation day 4, very few myotubes are observed, and by day 8, the fusion index is still significantly reduced for both KO1 and KO2 C2C12 cells. Scale bar, 125 µm. (*f*) Two shRNAs were designed to reduce the expression of STRN3. The shRNA#2 had the highest depletion in expression and was used for further experiments. Depletion of STRN3 did not impart C2C12 differentiation (*g*) or myotube formation (*h*). For (*a,b*), the RT-qPCR was performed in three technical replicates for each of the three biological replicates. For (*e*), the quantification of the fusion index on day 8 of differentiation was performed in many fields of each of the three biological replicates. The average of each biological replicate is plotted. *p < 0.05; **p < 0.01; ***p < 0.001; n.s., not significant.

Skeletal myogenesis is regulated by the myogenic regulatory factors (MRFs) comprising the basic helix–loop–helix transcription factors MyoD, Myf5, MyoG and MRF4 [3]. To assess any impact of SLMAP3 loss on the differentiation of C2C12 myoblasts, we investigated the expression of these MRFs. Real-time quantitative polymerase chain reaction (RT-qPCR) indicated downregulation of MyoD and MyoG (figure 3*a*), with MyoD and MyoG expression reduced ~9-fold and ~19-fold, respectively, due to SLMAP3 loss (figure 3*b,c*), which is similar to what we observed in SLMAP^−/−^ embryos. These results indicate that deletion of SLMAP3 in C2C12 myoblasts recapitulates transcriptional changes observed in SLMAP3^−/−^ embryos and represents an excellent model to investigate further the mechanisms of SLMAP3 action in myogenesis.

Given the changes in the MRF transcripts in SLMAP3^−/−^ C2C12 myoblasts, we proceeded to differentiation assays. For this, we incubated cells with 2% horse serum medium and collected protein lysates on days 0, 2 and 4 of differentiation for western blot analysis. The results show that both SLMAP3^−/−^ C2C12 cells have drastically reduced expression of MyoG and myosin heavy chain (MHC) proteins, although no changes in MyoD levels were observed on day 0 and day 2 of differentiation (figure 3*d*), despite the reduced transcripts observed in the RT-qPCR (figure 3*a*). On day 4, the KO cells still had significant MyoD expression, which is reflective of a defect in differentiation. A significantly reduced fusion index during differentiation of SLMAP3^−/−^ myoblasts was also notable by day 4 (figure 3*e*), and on day 8 of differentiation, it was reduced by ~93% in KO1 and ~40% in KO2 (figure 3*e*). These findings indicate that SLMAP3 is important for the proper skeletal myoblast differentiation and fusion via mechanisms involving the temporal expression of key myogenic factors such as MyoG and MyoD.

### 2.5. Depletion of STRN3, a STRIPAK component, does not affect myoblast differentiation

SLMAP3 is found in complex with STRIPAK together with STRN3, the latter that acts as a regulatory B‴ subunit of PP2A phosphatase [25,26]. Similarly to SLMAP3, STRN3 knockout results in embryonic lethality with complete penetrance [44]. Due to the partnership between SLMAP3 and STRN3 in the STRIPAK, and because of the lethal phenotype with their knockout, we questioned if depletion of STRN3 would also affect skeletal myoblasts. Two shRNAs targeting STRN3 and a shRNA scramble (SC) control were designed and packaged in lentivirus and used to transduce C2C12 myoblasts. Both the shRNAs reduced STRN3 protein levels, but shRNA STRN3#2 completely abolished its expression (figure 3*f*). Induction of myoblast differentiation with low serum levels showed that the C2C12-shRNA STRN3#2 cells did not have reduced expression of MyoD, MyoG or MHC (figure 3*g*), nor any impairment of myotube formation (figure 3*h*) after 5 days of differentiation compared to the C2C12-shRNA SC control. These findings suggest that SLMAP3 action in myoblast differentiation is distinctly unique from that of STRN3.

### 2.6. Differentiating SLMAP3^−/−^ myoblasts present defective MTOC recruitment to the nuclear envelope and reduced Golgi belt-like ring structure formation

Given that SLMAP3 can reside at the centrosome in association with its identified interactors AKAP9, PCNT, PCM1 and kinesin-1 subunits, we hypothesized that it contributes to the function of these proteins in myogenesis. To investigate this, we analysed the MTOC switch from the centrosome to the NE during the differentiation of SLMAP3^−/−^ C2C12 cells.

Firstly, we performed the differentiation of SLMAP3^−/−^ C2C12 myoblasts for 5 days and analysed the localization of pericentrin, AKAP6 and Kif5b proteins by immunofluorescence. AKAP6 was reported to be a key player in the MTOC switch of centrosomal proteins to the NE [18,21]. Because these proteins were described to localize in the NE after MyoG expression [21], we only analysed MyoG+ cells. With CellProfiler software, we used the MyoG staining to mask the cells and quantify their pericentrin, AKAP6 and Ki5fb mean intensity staining in concentric bands of 1 µm around the nucleus, which were designated NE, perinuclear (Peri) and cytoplasm (Cyto). It is notable that pericentrin, AKAP6 and Kif5b have the NE recruitment impaired in SLMAP3^−/−^ MyoG+ C2C12 cells (electronic supplementary material, figure S3*a–c*, respectively). The mean intensity of pericentrin, AKAP6 and Kif5b in the NE was 5.5-fold, 3.5-fold and 2.47-fold higher in the WT compared to the KO cells. Also, the differences in the mean intensity between NE and Cyto for their staining in SLMAP3^−/−^ MyoG+ C2C12 cells were reduced compared to WT controls, suggesting a reduced recruitment of these proteins to the NE.

**Figure 4.**
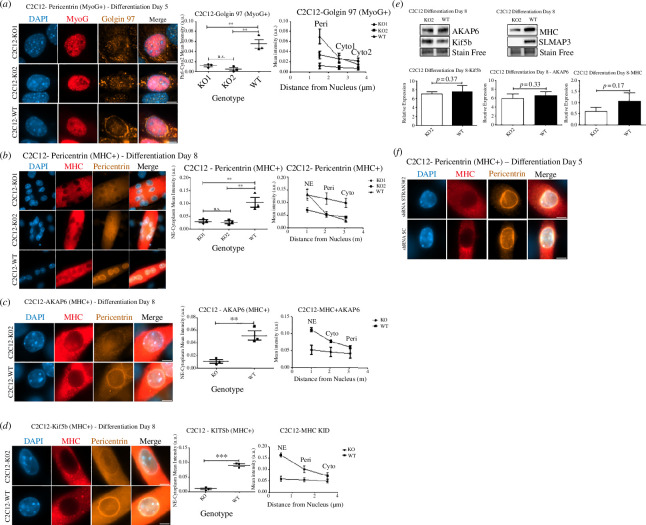
Differentiating SLMAP3^−/−^ myoblasts present defective MTOC recruitment to the NE and reduced Golgi belt-like ring structure formation. The recruitment of Golgi (*a*) to PEs is reduced in SLMAP3^−/−^ MyoG+ cells, as indicated by the mean intensities of its staining in PEs. In SLMAP3^−/−^ MHC+, the recruitment of pericentrin (*b*), AKAP6 (*c*) and Kif5b (*d*) to the NE after 8 days of differentiation is also impaired. (*e*) In differentiated myoblasts, the protein expression of AKAP6 and Kif5b in SLMAP3 KO is not significantly different from the control WT cells. After 8 days of differentiation, the MHC expression is no longer significantly reduced in SLMAP3 KO cells, although the tendency of reduced expression is notable. (*f*) Depletion of STRN3 did not affect the recruitment of pericentrin to the NE in MHC+ C2C12 cells after 5 days of differentiation. Scale bar, 8 µm. For (*a–d*), multiple fields for each of the three biological replicates were analysed. The average of each biological replicate is plotted. For (*e*), three biological replicates for each genotype were analysed. **p < 0.01; ***p < 0.001; n.s., not significant.

If pericentrin, AKAP6 and Kif5b recruitment to the NE is affected, then a defective remodelling of the Golgi apparatus to PEs is also expected. AKAP6 was reported to be recruited to the NE by the interaction with the LINC complex member Nesprin-1α [18,21,45]. AKAP6 binds to AKAP9, which in turn is important for Golgi recruitment [18]. To assess the Golgi remodelling, we measured the intensity of the staining Golgi around the nucleus of MyoG+ cells, with the modification of having the first band starting 0.5 µm further from the NE, and then calling the resulting bands as Peri, Cyto1 and Cyto2. The recruitment of Golgi apparatus to PEs was reduced in SLMAP3^−/−^ myoblasts as well, with a higher difference between the mean intensity in the Peri and Cyto2 bands in the WT compared to the KO cells (figure 4*a*).

To test if the reduced recruitment of centrosomal proteins to the NE of SLMAP3^−/−^ myoblasts was because of either a delayed differentiation or an anchorage defect, we performed the pericentrin, AKAP6 and Kif5b analysis again, but in MHC+ cells after 8 days of differentiation. Remarkably, their recruitment to the NE was still significantly impaired in SLMAP3^−/−^ MHC+ cells (figure 4*b–d*). To further validate this finding, we analysed pericentrin recruitment to the NE in MHC+ cells in two other colonies generated by each sgRNA, and the same phenotype was observed (electronic supplementary material, figure S3*d*). To rule out the possibility of reduced expression of these proteins due to the differentiation defects, we performed western blot with C2C12 lysates after 8 days of differentiation, and the expressions of AKAP6 and Kif5b were not significantly different from that of the control WT cells (figure 4*e*). Additionally, after 8 days of differentiation, the MHC expression in C2C12 KO2 cells was no longer significantly reduced compared to the WT (figure 4*e*). Therefore, the reduced presence of AKAP6, Kif5b and most likely pericentrin in the NE is due to protein mislocalization and not changes in their protein levels.

Although depletion of STRN3 did not affect C2C12 differentiation or myotube formation, we tested if this protein had any impact on the recruitment of MTOC proteins to the NE. After 5 days of differentiation, the STRN3-depleted cells did not present defective pericentrin recruitment to the NE (figure 4*f*).

These findings suggest SLMAP3 has a unique mode of action in muscle formation through a structural mechanism involving the anchorage of centrosomal proteins to the NE, and consequently, for the Golgi remodelling in PEs and myoblast differentiation, a function that is distinct from STRN3.

### 2.7. Differentiating SLMAP3^−/−^ myoblasts display defective microtubule growth from the nuclear envelope

**Figure 5.**
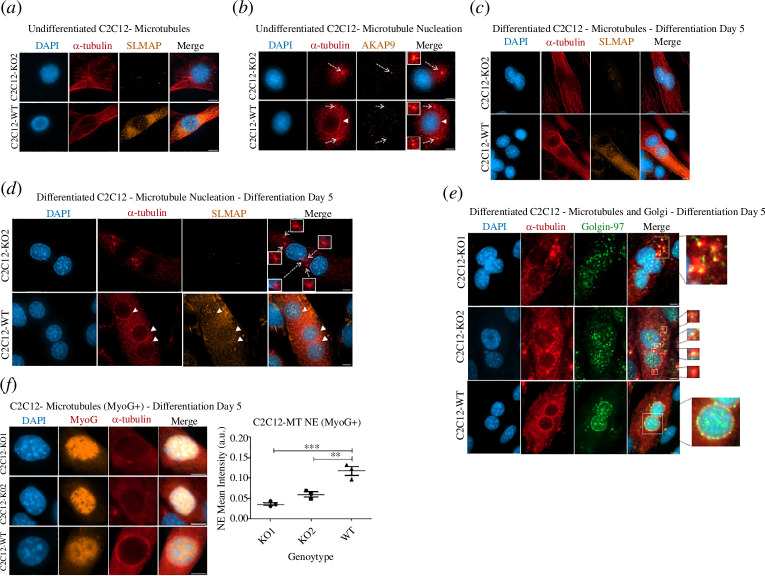
Differentiating SLMAP3^−/−^ myoblasts display defective microtubule growth from the NE. (*a*) Microtubule cytoskeleton in undifferentiated C2C12 cells. No obvious abnormalities could be seen in SLMAP3^−/−^ myoblasts. (*b*) In undifferentiated cells, microtubule nucleation assay shows a stronger microtubule growth from the centrosomes of SLMAP3^−/−^ myoblasts (arrow), whereas microtubule nucleation in WT cells seems already partially switched to the NE (arrowhead). (*c*) Microtubule cytoskeleton in differentiated C2C12 cells. Parallel arrays of microtubules are present in both SLMAP3^−/−^ and WT cells. (*d*) Microtubule nucleation assay in differentiated C2C12 cells shows that in WT cells, the microtubules mostly grow from the NE (arrowhead), whereas in SLMAP3^−/−^ cells the growth is from sites in the cytoplasm (arrows). (*e*) Microtubule nucleation assay in differentiated C2C12 cells shows that the sites of microtubule growth in the cytoplasm in SLMAP3^−/−^ cells correspond to fragments of the dispersed Golgi (sites of microtubule nucleation from the Golgi expanded from the merged images for clarity). (*f*) The quantification of the mean intensity of microtubules in the NE of MyoG+ myoblasts confirms the reduced microtubule growth from this site in SLMAP3^−/−^ cells. Scale bar, 8 µm. For the quantification, multiple fields of each of the three biological replicates of each sample were analysed. The average of each biological replicate is plotted. **p < 0.01; ***p < 0.001.

Analysis of microtubule cytoskeleton in both SLMAP3^−/−^ and WT cells before differentiation does not indicate any obvious differences between them (figure 5*a*). Also, microtubule nucleation assay with α-tubulin and AKAP9 staining shows centrosomal microtubule nucleation in both undifferentiated SLMAP3^−/−^ and WT cells (figure 5*b*). However, it is observable that microtubule nucleation from the centrosome in SLMAP3^−/−^ cells is stronger compared to WT myoblasts (arrows in figure 5*b*). This could be because in the WT cells, there was already a level of decentralization of MTOC activity from the centrosome to the NE (arrowhead in figure 5*b*). In SLMAP3^−/−^ fibroblasts, the microtubule cytoskeleton and Golgi (electronic supplementary material, figure S4*a*) and microtubule growth from the centrosome (electronic supplementary material, figure S4*b*) do not have any obvious abnormalities. Additionally, microtubule nucleation remains in the centrosome of fibroblasts rather than decentralized to the Golgi (electronic supplementary material, figure S4*c*). This is consistent with our findings in undifferentiated C2C12 myoblasts, where centrosomal MTOC activity is not reduced in cells lacking SLMAP3.

Next, we asked how the microtubule growth would appear in SLMAP3^−/−^ C2C12 cells after differentiation, considering their defective MTOC switch to the NE. The initial visualization of the microtubule cytoskeleton in myotubes after 5 days of differentiation shows that parallel arrays of microtubules along the myotube structure are present in both SLMAP3^−/−^ and WT myoblasts (figure 5*c*). To visualize the sites of microtubule growth, we performed a microtubule nucleation assay. It is visible that the microtubule growth in SLMAP3^−/−^ myotubes takes place in the cytoplasm (figure 5*d*). By performing this same assay but with Golgin-97 staining, it became evident that those sites of microtubule nucleation in the cytoplasm correspond to the dispersed Golgi (figure 5*e*). The quantification of the mean intensity of α-tubulin staining in the NE was also significantly reduced in SLMAP3^−/−^ MyoG+ cells (figure 5*f*), suggesting reduced microtubule nucleation from the NE in differentiation.

These results suggest two things: firstly, the nucleation of the microtubules from the NE in SLMAP3^−/−^ myotubes is compromised, consistent with our findings of the defective MTOC switch; secondly, the microtubule nucleation from the centrosome before differentiation and from the Golgi after differentiation is unaffected. Thus, in the context of skeletal myogenesis, SLMAP3 is important for microtubule nucleation specifically from the NE.

### 2.8. Skeletal muscle of SLMAP3^−/−^ embryos display defective recruitment of pericentrin to the nuclear envelope and nuclear mispositioning

**Figure 6.**
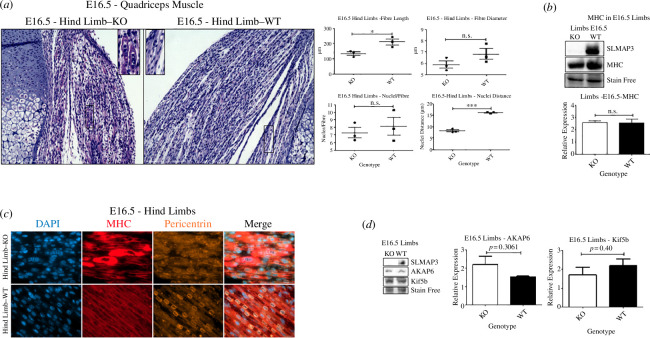
Skeletal muscle of SLMAP3^−/−^ embryos display defective recruitment of pericentrin to the NE and nuclear mispositioning. (*a*) H&E staining of E16.5 quadriceps indicates that SLMAP3^−/−^ embryos have abnormal muscle fibres, with reduced length and reduced internuclear distance. Fifteen fibres analysed across the quadriceps of three animals of each genotype. The averages corresponding to each animal are plotted. (*b*) Western blot analysis of E16.5 limbs shows no difference in MHC expression in SLMAP3^−/−^ embryos compared to WT. (*c*) Pericentrin and MHC staining in muscle quadriceps indicate that skeletal muscle fibres of SLMAP3^−/−^ embryos have defective NE-MTOC. (*d*) The protein expression of AKAP6 and Kif5b in SLMAP3 KO limbs is not significantly different from the WT samples. Scale bars, (*a,e*) 200 µm and (*c*) 20 µm. *p < 0.05; ***p < 0.001; n.s., not significant.

Given the downregulation of genes involved in muscle development in SLMAP3^−/−^ embryos and the defects in differentiation, myotube formation and MTOC switch, we analyed sections of hind limbs at E16.5, a time when secondary myogenesis is taking place and sustained mostly by myoblast fusion [3]. It is notable that the overall muscle in the SLMAP3^−/−^ embryos, visualized by haematoxylin and eosin (H&E) staining, is smaller compared to WT (figure 6*a*). We manually quantified the fibre length across the muscle using Fiji software and found that the fibre length in SLMAP3^−/−^ embryos was reduced by approximately 40% when compared to WT (figure 6*a*). Although we could not identify significant changes in the diameter and in the number of nuclei per fibre of the SLMAP3^−/−^, we found that the inter-nuclear distance within the fibres was approximately half of the distance in WT embryonic muscle (figure 6*a*). In one SLMAP3^−/−^ embryo, multiple muscle fibres show agglomeration of nuclei (figure 6*a*), compared with the even spread seen in WT. These findings suggest that the mechanism of muscle abnormalities in SLMAP3^−/−^ embryos involves, at least partially, an impairment of nuclei positioning.

The fact that we could not identify differences in the number of nuclei per fibre in SLMAP3^−/−^ embryos could be an indication that myoblast fusion was not as impaired as we observed in culture during differentiation of C2C12 myoblasts. Also, although the overall muscle is smaller and the inter-nuclei distance is reduced in SLMAP3^−/−^ embryos, the muscle fibres are visibly formed (figure 6*a*). Additionally, at E16.5, the analysis of MHC expression in the limbs by western blot indicates no difference in SLMAP3^−/−^ embryos compared to WT (figure 6*b*), despite the observed downregulation of skeletal muscle genes in these animals at E11.5. It could be that the muscle development in SLMAP3^−/−^ embryos is delayed, but it reaches the formation of muscle fibres by E16.5 through endogenous compensatory mechanisms that are absent in tissue culture. It is also plausible that temporal expression of other SLMAP isoforms may compensate for SLMAP3 deficiency in the various fibre types [31,32].

Since SLMAP3 appears to be involved in the centrosomal MTOC switch to the NE in cultured myotubes, we investigated quadriceps muscles of SLMAP3^−/−^ embryos to see if a similar MTOC switch was evident *in vivo*. Immunofluorescence staining indicated that pericentrin could be observed in the NE of MHC+ tissue, but this staining was weaker and much less defined in SLMAP3^−/−^ muscles compared to WT (figure 6*c*). These data are similar to that seen in cultured myotubes derived from SLMAP3^−/−^ C2C12 myoblasts (figure 4*b*). We also analysed the protein expression of AKAP6 and Kif5b in the limbs of E16.5 embryos lacking SLMAP3, and no differences were seen compared to the WT (figure 6*d*), which is consistent with our observations in C2C12 derived myotubes (figure 4*e*).

Collectively our data imply that loss of SLMAP3 in C2C12 myoblasts or *in vivo* negatively impacts muscle development at the level of NE-MTOC dynamics.

### 2.9. Differentiating SLMAP3^−/−^ myoblasts have abnormal localization of LINC complex proteins

**Figure 7.**
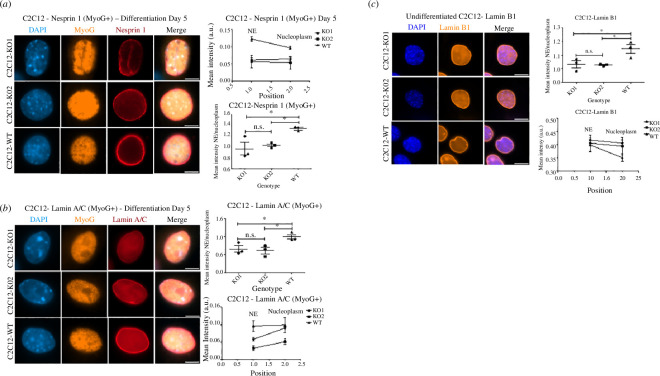
Differentiating SLMAP3^−/−^ myoblasts have abnormal localization of LINC complex proteins. The LINC complex members Nesprin-1 (*a*) and Lamin A/C (*b*) have defective recruitment to the NE in SLMAP3^−/−^ MyoG+ myoblasts, as indicated by the ratio between their mean intensity in NE and nucleoplasm. (*c*) Staining of Lamin B1 indicates the subtle difference between SLMAP3^−/−^ and WT cells. Scale bar, 8 µm. Multiple fields of each of the three biological replicates of each sample were analysed. The average of each biological replicate is plotted. *p < 0.05; n.s., not significant.

Given our data that the MTOC switch is critically dependent on SLMAP3 for myogenesis, we assessed if the localization of Nesprin-1 was also affected in differentiating MyoG+ myoblasts. Kinesin-1 was reported to be important for nuclei positioning in myoblasts, and it is recruited to the NE by Nesprin-1α [10,20,35–37,46,47]. Nesprin-1α is also responsible for the recruitment of AKAP6 [18,21,45]. Since SLMAP3 appears in complex with centrosomal proteins that are mislocalized in SLMAP^−/−^ myoblasts, we examined any changes in anchorage of Nesprin-1 in the NE by comparing its mean intensity of staining between the NE band and distribution in the nucleoplasm. Surprisingly, the Nesprin-1 mean intensity in the NE was reduced in the SLMAP3^−/−^ MyoG+ cells (figure 7*a*). If the lack of SLMAP3 was affecting solely the MTOC proteins and Kinesin-1, we would expect an intact Nesprin-1 staining, which does not seem to be the case.

Since Nesprin-1, a LINC complex member [48], was affected in the myoblasts lacking SLMAP3, we asked if lamins would also be changed in these cells. Lamins are intermediate filaments that decorate the inner side of the NE, and lamin A/C interacts with Nesprin-1α [48]. Lamins B1 and B2 have a constitutive expression in all cells and are encoded by two different genes, while lamin A/C are encoded by the single gene *lmna* and expressed only in differentiated cells [48]. For lamin A/C we analysed only MyoG+ cells since it has been demonstrated that myoblasts at this stage of differentiation already display higher lamin A/C recruitment from the nucleoplasm to the NE [49,50]. Indeed, the NE staining of lamin A/C was reduced in SLMAP3^−/−^ MyoG+ cells when compared to the WT myoblasts (figure 7*b*). The NE recruitment of the constitutive lamin B1 was only slightly changed in SLMAP3^−/−^ cells before differentiation (figure 7*c*). These results suggest that the lack of SLMAP3 also impacts the LINC complex and is important for the proper recruitment of Nesprin-1 and lamins to the NE in myoblasts.

### 2.10. SLMAP3 is crucial for developing muscle independent of Hippo signalling

**Figure 8.**
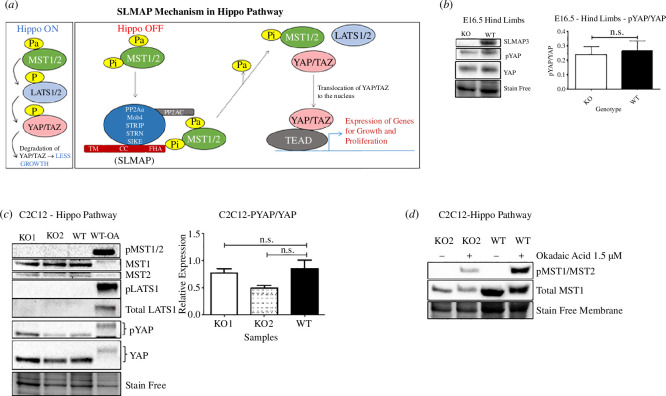
SLMAP3^−/−^ C2C12 cells and skeletal muscles do not present upregulated Hippo signalling. (*a*) Diagram summarizing the proposed mechanism of SLMAP3 in repressing the Hippo pathway by recruiting MST1/2 kinases to STRIPAK, where they are deactivated [29,30]. Pa = activating phosphorylation; Pi = inhibitory phosphorylation. (*b*) Hippo signalling is not affected in the hind limbs of SLMAP3^−/−^ E16.5 embryos since no changes in YAP phosphorylation were detected. (*c*) No significant changes in the Hippo pathway are observed in SLMAP3^−/−^ C2C12 myoblasts, (*d*) even in the presence of the PP2A inhibitor okadaic acid.

SLMAP3 has been reported to be part of the STRIPAK complex [25,26], in an evolutionary conserved way [39,51], and proposed to negatively regulate the Hippo pathway (figure 8*a*), which consists of a cascade of kinases leading to the phosphorylation of the transcriptional coactivators YAP and TAZ, resulting in their cytoplasm sequestration and degradation. In the proposed mechanism, SLMAP3 recruits the serine/threonine sterile-20-like kinases MST1/2, to STRIPAK, where they are dephosphorylated and deactivated by PP2A phosphatase. The depletion of SLMAP3 could then result in higher activation of MST1/2, reducing the nuclear localization of YAP and TAZ and, consequently, decreasing the expression of genes associated with growth, proliferation and survival [27–30].

Due to the observed skeletal muscle abnormalities in SLMAP3 KO embryos, we analysed YAP phosphorylation in E16.5 limbs. No significant differences were detected between SLMAP3 KO and WT embryos (figure 8*b*). Further analysis of YAP and MST1/2 phosphorylation in C2C12 myoblasts does not indicate any changes in SLMAP3 KO myoblasts either, even with okadaic acid treatment to inhibit PP2A (figure 8*c,d*). Furthermore, the RNA-seq did not reveal any impact on Hippo-regulated genes due to SLMAP3 loss. Together, these results strongly suggest that the defective muscle development due to SLMAP3 loss is independent of any involvement of Hippo signalling. Our data also indicated that STRN3, which is a key regulator of Hippo, was without effect on myotube formation or the NE-MTOC.

## 3. Discussion

We established that SLMAP3 is critical for the formation of muscle through mechanisms that involve the positioning of the nuclei and the switch of MTOC components from the centrosome to the NE during myogenesis. SLMAP3 is structurally designed to target the MTOC and NE; thus the impairment in MTOC dynamics due to SLMAP3 loss could be a result of defective attenuation of centrosomal activity, i.e. reduced microtubule nucleation from the centrosome and/or failure in the anchoring of the MTOC machinery to the NE. The newly identified interactors of SLMAP3 (AKAP9, PCNT, PCM1 and Kinesin-1 subunits) that are MTOC components further support the role of SLMAP3 in the positioning of centrosomal proteins. The impact of SLMAP3 loss on muscle-specific gene expression as well as nuclei mispositioning in developing myofibres point to a novel role at the NE in connecting cytoskeletal changes to nuclear function.

**Figure 9.**
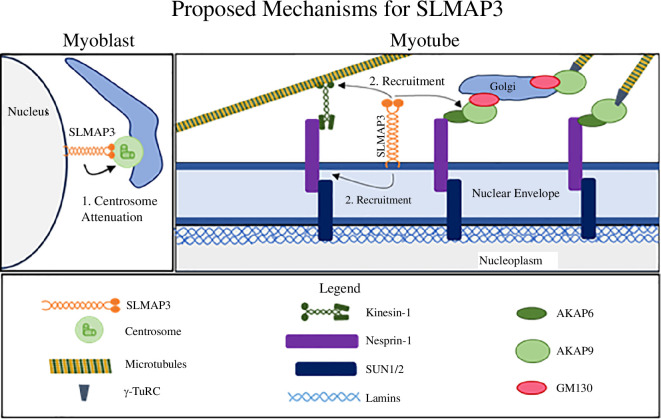
Model for SLMAP3 action in myogenesis. SLMAP3 targets the NE and the centrosome to guide the MTOC switch from the centrosome to the NE during myogenesis. Its loss could either impact centrosome attenuation (reduction of centrosomal microtubule nucleation) and/or the distribution of centrosomal proteins to the NE. SLMAP3 binds to the centrosomal components (pericentrin, AKAP9 and kinesin-1 subunits) and its loss impairs the recruitment of these proteins and AKAP6 to the NE during skeletal myogenesis, reducing microtubule nucleation from this organelle. Consequently, this disrupts the formation of the Golgi belt-like ring structure since AKAP6 and AKAP9/pericentrin bridge Nesprin-1 and Golgi through GM130 [18,20,21]. Furthermore, loss of SLMAP3 disrupts the nucleoskeleton, affecting the recruitment of Nesprin-1 and potentially impacting lamins and gene expression.

In microtubule nucleation assays, we observed microtubules growing from the Golgi of SLMAP3^−/−^ myoblasts upon differentiation. It is tempting to assume that some level of centrosome attenuation was occurring to increase the MTOC activity in the Golgi instead of NE. In RPE1 cells, laser ablation of centrosome induces microtubule nucleation in the Golgi, suggesting that the excess of MTOC proteins go to this organelle [52]. However, we should consider that: firstly, the microtubule growth colocalizing with Golgi in SLMAP3^−/−^ cells could correspond to centrosomes from the fused cells that failed to migrate to the NE. Clustering of centrosomes from fused cells is observed in osteoclasts [53]. Secondly, in the context of skeletal myogenesis, the microtubule growth from the Golgi could be independent of centrosome attenuation. Therefore, we suggest that SLMAP3 could be important for the attenuation of centrosome activity, as modelled in figure 9, where SLMAP3 interacts with centrosomal components via its N-terminal FHA domain and targets the NE through the C-terminal hydrophobic tail anchor. We know it is not important for microtubule nucleation because the centrosomal microtubule growth in SLMAP3^−/−^ myoblasts was not impacted, and SLMAP3 is not associated with the Golgi [23,38,40], an organelle known to have microtubule nucleation activity [54–57].

Our data that the loss of SLMAP3 impacted the distribution of MTOC components at the NE during differentiation further imply a mechanism where SLMAP3 contributes to the anchorage of MTOC proteins at the NE (figure 9). In addition, the recruitment of Nesprin-1, which is not an MTOC protein and therefore independent of centrosomal attenuation, was also affected at the NE in differentiating SLMAP3^−/−^ myoblasts (figure 9). We noted a reduction in lamin A/C at the NE with redistribution in the nucleoplasm in SLMAP3^−/−^ myoblasts, which supports a role for SLMAP3 in organizing the nuclear cytoskeleton. In this regard, our data here also suggest interactions of SLMAP3 with nuclear lamins, indicating that SLMAP could potentially reside at the nuclear face of the NE to impact gene activity, although this remains to be tested.

The implication of defective recruitment of centrosomal proteins to the NE during skeletal myogenesis is nuclei mispositioning, a feature observed in patients with EDMD and CNM [11,12]. In EDMD, it is usually caused by mutations in genes encoding proteins from the LINC complex, such as emerin, SUN1, SUN2, lamin A/C and Nesprin-1 and Nesprin-2 [14,48,58], characterized by progressive muscle weakness and wasting, early contractures and cardiac abnormalities [13,14]. In differentiating myoblasts, the NE up to the Golgi is organized as follows: in the LINC complex, Nesprin-1 and Nesprin-2 members localize in the outer nuclear membrane (ONM) [48], although their smaller isoforms can also localize in the inner nuclear membrane (INM) [59–61]. In the NE lumen, the KASH domain of Nesprin proteins interacts with the SUN domain proteins, which are anchored in the INM [46]. In the INM, both the SUN proteins and the smaller Nesprin isoforms interact with lamin A/C and emerin [48,62]. Additional proteins are recruited to the ONM by interaction with Nesprin-1α, a smaller isoform of Nesprin-1. These include the motor proteins from the dynein and kinesin-1 complexes [10,20,36,37,47] and the protein AKAP6 [18,21,45]. Another level of organization is the binding of the paralogues AKAP9 and pericentrin to AKAP6 [18,21]. The Golgi belt-like ring structure around the nucleus is then tethered by AKAP9 [18]. In this organization, microtubules could be nucleated from both NE and Golgi [17,18].

We found that SLMAP3 interacts with the outer layer proteins, such as Kinesin-1 and centrosomal proteins. However, because we observed abnormalities in the distribution of all these proteins in SLMAP3^−/−^ myoblasts, SLMAP3 may also be important for the proper localization of the LINC complex. Our finding that SLMAP3 interacts with proteins of the nuclear cytoskeleton implies that SLMAP3 could integrate the MTOC-NE dynamics via its structural domains. The alternative hydrophobic C-terminal sequences in SLMAP3 would make it an ideal candidate to target both outer and INMs to orient the protein so as to serve functions across the NE thus integrating centrosomal activity with the nuclear lamina, although this needs to be tested.

Depletion or mutation of LINC complex members, AKAP6 and Kinesin-1, have been reported to lead to differentiation and myotube formation defects [47,49,63–68] (electronic supplementary material, table S3) much like that seen here with SLMAP3 loss, potentially indicative of a common pathological mechanism. Changes in emerin and lamin A/C can lead to changes in the tethering of heterochromatin in the NE and impact the expression of genes of the myogenic programme [61,69]. It is conceivable that loss of SLMAP3 leading to the redistribution of lamins at the NE may also impact chromatin dynamics and lead to the observed changes in muscle gene expression.

Although SLMAP3^−/−^ myoblasts presented a reduced fusion index and decreased expression of MyoG and MHC, SLMAP3^−/−^ embryos at E16.5 did not indicate any changes in MHC expression. E11.5 embryos showed a decrease in the expression of genes involved in muscle development, and the muscle from SLMAP3^−/−^ E16.5 embryos was clearly abnormal, with reduced fibre size and inter-nuclei distance. Differentiation defects have been reported in culture for mutations in lamin A/C and emerin [49,63–67], while mice null for either of them are born without observable muscle defects [70,71]. Lamin A/C KO animals become dystrophic a few weeks after birth [68], and the emerin KO mice have defective muscle regeneration [71]. Therefore, skeletal muscle differentiation problems in SLMAP3^−/−^ animals could become even more evident in the context of muscle regeneration.

In our analysis of the microtubule cytoskeleton of differentiated SLMAP3^−/−^ myoblasts, we noticed that parallel arrays of microtubules were present. However, with microtubule nucleation assay we could see that microtubules were growing from sites colocalizing with Golgi. The presence of parallel arrays of microtubules even when the NE-MTOC is impaired seems to be a common feature, since it was observed in differentiated myoblasts with knockout or depletion of Nesprin-1α, Sun1, Sun2, Sun1/2, Kif5b and AKAP6 [10,20,21,36,72].

The fact that SLMAP3 as a STRIPAK component was found to be crucial in the NE-MTOC formation suggests that other members of the complex could also play a role in this process. However, the depletion of STRN3 had no impact on NE-MTOC or myogenesis. Whether other paralogues STRN or STRN4 could serve compensatory roles needs consideration, although the knockout of either STRN3 or STRN is enough to result in embryonic lethality.

Finally, despite the mechanistic descriptions of SLMAP3 as a negative regulator of the Hippo pathway [27–30], changes in this signalling cascade could not be detected in the limbs of SLMAP3^−/−^ embryos or SLMAP3^−/−^ myoblasts. In myoblasts, YAP was reported to be important in the early proliferative phase, but its cytoplasmic sequestration is essential for terminal differentiation [73]. In human cardiomyoblasts, the activation of MST and LATS kinases was sufficient to induce centrosome disassembly and relocation of PCM proteins to the NE [74]. If loss of SLMAP3 was inducing Hippo, then we would expect no defects in the terminal differentiation *in vivo* or in myoblasts. Therefore, the defects observed in SLMAP3 deficiency are most likely independent of Hippo and point to novel mechanisms involving the newly identified interactors of SLMAP3, including AKAP9, Kinesin-1 and centrosomal components. It is notable that STRN3 loss had no impact on the MTOC or myotube development implying that distinct STRIPAK components may serve unique roles in a cell-/tissue-specific manner as exemplified here by SLMAP3.

## 4. Conclusion

Here we identify SLMAP3 as an essential player in developing skeletal muscle by participating in positioning nuclei and the formation of MTOC at the NE to impact the differentiation programme. Our data define a novel role for SLMAP3 in the formation of non-centrosomal MTOCs and link to nuclear activity and tissue development. The ability of SLMAP3 to assemble with components of the MTOC and the nuclear membrane makes it an ideal player to serve such functions which appear to be independent from any involvement with Hippo signalling.

## 5. Material and methods

### 5.1. Transgenic mice generation

The generation of the flox-SLMAP mouse line was already described in detail [31]. The Cre transgenic mouse was a gift from Dr Nemer Mona, University of Ottawa. For the genotyping of both mouse lines and the breeding strategy, we followed the procedures already described [31]. The mice in this study were housed at the Animal Care and Veterinary Service (ACVS) Barrier Facility at the University of Ottawa and handled in compliance with the Canadian Council on Animal Care, Guide to the Care and Use of Experimental Animals, 2 vols. (Ottawa, Ont.: CCAC, 1980−1993) and Animals for Research Act, R.S.O. 1990, c.A. 22. The protocols for animal study were approved by the Animal Care Committee at the University of Ottawa.

### 5.2. Cell culture and treatment conditions

MEFs were isolated from E14.5 embryos as described [75], and tails were used for genotyping. All WT MEFs used are from littermates of SLMAP3^−/−^ embryos. The C2C12 myoblast cell line was commercially provided by the American Type Culture Collection. Cells were kept in humidified atmosphere with 5% CO_2_ and at 37°C. For MEFs, we used a medium containing high glucose Dulbecco’s Modified Eagle Medium (DMEM) (Wisent, cat. 319-005), 1× MEM (Gibco, cat. 11140050), 20 mM HEPES, 10% fetal bovine serum (FBS; Wisent, cat. 080-150), 1× antibiotic-antimycotic (Gibco, cat. 15240062) and 0.1 mM β-mercaptoethanol, as described [76]. For C2C12 cells, we used a medium containing DMEM (Wisent, cat. 319-005), 10% FBS (Wisent, cat. 080-150) and 1× antibiotic-antimycotic (Gibco, cat. 15240062). The C2C12 differentiation medium was prepared with DMEM without sodium pyruvate (Wisent, cat. 319-015), 1× antibiotic-antimycotic (Gibco, cat. 15240062), 25 mM HEPES and 2% horse serum (Gibco, cat. 16050122). We considered ‘differentiating myoblasts’ all the cells incubated with differentiation medium. For okadaic acid (Cell Signaling, cat. 5934) condition, we used a final concentration of 1.5 µM for 1 h of treatment. For transfections, we used Lipofectamine 3000 (Invitrogen, cat. L3000001) following the protocol of the manufacturer. Microtubule nucleation assays were performed in cells as described elsewhere [77], with the final nocodazole concentration of 8.3 μM and incubation time of 2 h at 37°C. After nocodazole washout in cold medium, coverslips were kept in the saponin solution at 37 ͦ C for 45 s, followed by incubation with medium at 37 ͦ C for 50 s. Cells were subsequently fixed in freezer-cold methanol for 5 min.

### 5.3. SDS–PAGE and western blot

Protein lysates were extracted on ice from cells by cell scraping and by homogenization of snap-frozen tissue. The lysis buffer contained 20 mM Tris (pH 7.5), 150 mM NaCl, 1 mM EDTA, 1 mM EGTA and 1% Triton X-100. For each 10 ml of buffer, one tablet of Pierce Protease Inhibitor (Thermos Scientific, cat. A32955) and of Pierce Phosphatase Inhibitor (Thermo Scientific, cat. A32957) were used. Proteins were denatured with SDS buffer containing 10% glycerol, 2% SDS, 62.5 mM Tris–HCl (pH 6.8) and 10% β-mercaptoethanol and boiled for 5 min. The lysates were loaded on sodium dodecylsulfate polyacrylamide gel electrophoresis (SDS–PAGE) gel and posteriorly transferred to polyvinylidene fluoride membrane (Bio-Rad). For membrane wash, blocking and antibody solutions, we used Tris-buffered saline (TBS-T) buffer containing 1 M Tris, 290 mM NaCl and 0.1% Tween 20 (pH 7.4). Antibody solutions were prepared with 5% milk in TBS-T or with 5% BSA for primary antibodies targeting phosphorylated amino acid residues. Images were acquired on the Bio-Rad ChemiDoc system and analysed by densitometry on Image Lab Software (Bio-Rad). The list of the antibodies used for western blot (WB) is as follows:

**Table:** 

primary antibodies				
**target**	**company**	**catalogue**	**host**	**dilution WB**
SLMAP	Novus Biologicals	NBP1-81397	rabbit	1:500
SLMAP	Novus Biologicals	NBP1-81398	rabbit	1:1 000
STRN3/SG2NA (S68)	Novus Biologicals	NBP74572	mouse	1:500
MST1 (KRS2) (H-8)	Santa Cruz	SC-515051	mouse	1:200
MST2 (KRS1) (87.K)	Santa Cruz	SC-130405	mouse	1:200
phospho-MST1 (Thr183)/MST2 (Thr180) (E7U1D)	Cell Signaling	49332	rabbit	1:500
YAP (1A12)	Cell Signaling	12395	mouse	1:1 000
phospho-YAP (Ser127)(D9W2I)	Cell Signaling	13008	rabbit	1:1 000
MyoD (G-1)	Santa Cruz	SC-377460	mouse	1:100
MyoG (F5D)	Santa Cruz	SC-12732	mouse	1:100
MHC	Developmental Studies Hybridoma Bank	MF20	mouse	1:50
lamin B1	Abcam	ab16048	rabbit	1:200
vimentin (D21H3) XP	Cell Signaling Technology	5741	rabbit	1:1 000
Akap6	Millipore Sigma	HPA048741	rabbit	1:100
Kif5B [EPR10276(B)]	Abcam	ab167429	rabbit	1:100

**Table:** 

secondary antibodies				
**target**	**company**	**catalogue**	**host**	**dilution WB**
peroxidase AffiniPure goat anti-rabbit IgG (H + L)	Jackson ImmunoResearch	111-035-144	goat	1:10 000
peroxidase AffiniPure goat anti-mouse IgG (H + L)	Jackson ImmunoResearch	115-035-146	goat	1:10 000

### 5.4. RT-qPCR

Primers were designed using the Primer-Blast tool (NIH) and validated with standard curve and band size of PCR products from cDNA of C2C12 cells. RNAs were extracted from cells with RNeasy Mini Kit (cat. 74104) from Qiagen according to the protocol of the manufacturer. We synthesized cDNA from the RNAs with the iScript Reverse Transcription Supermix for RT-qPCR from Bio-Rad (cat. 1708840). RT-qPCR was performed with the FastStart Universal SYBR Green Master (Rox; Millipore Sigma, cat. 4913850001). Glyceraldehyde-3-phosphate dehydrogenase (GAPDH) was used as the housekeeping gene. The samples were read on a Bio-Rad CFX 96 thermal cycler, and the data were analysed on Bio-Rad CFX Maestro. The primers used are listed below:

**Table:** 

primers	forward (5′−3′)	reverse (5′−3′)
Myf5	CTATTACAGCCTGCCGGGAC	CTCGGATGGCTCTGTAGACG
Mrf4	CCCCACAGATCGTCGGAAAG	CAGAATCTCCACCTTGGGCA
MyoG	CAGCCCAGCGAGGGAATTTA	AGAAGCTCCTGAGTTTGCCC
MyoD	ATAGACTTGACAGGCCCCGA	GCAGGTCTGGTGAGTCGAAA
Pax3	TCGAGAGAACCCACTACCCA	CCCCCGGAATGAGATGGTTG
GAPDH	GGTTGTCTCCTGCGACTTCA	TGGTCCAGGGTTTCTTACTCC

### 5.5. RNA sequencing

Total RNA was isolated from E11.5 whole embryos by RNeasy Fibrous Tissue Mini Kit according to the manufacturer protocol (Qiagen). The samples were then submitted to the StemCore Laboratories Genomics Core Facility at the University of Ottawa for RNA sequencing as follows: total RNA (1 µg) was quantified using a Qubit (Thermo Scientific), and its integrity was assessed on an AATI Fragment Analyzer (Agilent Technologies). Library construction was performed using TruSeq RNA v2 Library Preparation Kit (Illumina). Following library qualilty assurance/quality control (QA/QC) (as above for input), the eight libraries were pooled on a NextSeq 500 75 cycle High Output Flow cell with 1% PhiX spike-in. RNA-seq post-processing and differential expression analysis were prepared by the Ottawa Bioinformatics Core Facility. Briefly, reads were assigned to the GRCm38_GENCODE.vM19 transcriptome model using Salmon [78], and the differential expression of transcripts was analysed by DESeq2 [79]. The data discussed from this study have been deposited in NCBI’s Gene Expression Omnibus [80,81] and are accessible through GEO Series accession number GSE230748 (https://www.ncbi.nlm.nih.gov/geo/query/acc.cgi?acc=GSE230748). The data were validated by RT-qPCR, with the analysis of the expression of myod1, myog, myf5, myf6 and pax3.

### 5.6. Immunoprecipitation–mass spectrometry

Immunoprecipitation of SLMAP3 was performed on 75 µg of E12.5 mouse brain WT lysates by anti-SLMAP (Novus, cat. NBP1-81397) with Pierce™ Protein A/G Magnetic Beads (Thermo Fisher, cat. 88802) based on the manufacturer protocol. Immunoprecipitation of anti-IgG (Protein Tech, cat. 30000-0-AP) was performed identically and simultaneously as a control to identify non-specific interactions. The resulting samples were submitted to the Montreal Clinical Research Institute for MS as follows: pulled-down lysates incubated with anti-SLMAP and anti-IgG were treated with acetone to precipitate and purify approximately 20 µg of pellet. Protein extracts were then re-solubilized in 6 M urea buffer and 100 mM ammonium bicarbonate, followed by reduction with reduction buffer (45 mM DTT and 100 mM ammonium bicarbonate). Finally, samples were alkylated in 100 mM iodoacetamide and 100 mM ammonium bicarbonate. After reducing the urea concentration to 2M, 20 ng µl^−1^ of trypsin (Promega) was added to each sample, and digestion was performed at 37°C overnight. Prior to liquid chromatography with tandem mass spectometry (LC–MS/MS), protein digests were re-solubilized in 0.2% formic acid. Desalting/cleanup of the digests was performed using C18 ZipTip pipette tips (Millipore). Dried eluates were reconstituted in 2% acetronile/1% formic acid and loaded into a 75 μm i.d. × 150 mm Self-Pack C18 column installed in the Easy-nLC II system (Proxeon Biosystems). The buffers used for chromatography were 0.2% formic acid (buffer A) and 90% acetonitrile/0.2% formic acid (buffer B). The high performance liquid chromatography (HPLC) system was coupled to Orbitrap Fusion mass spectrometer Xcalibur 4.0 and Tune 2.0 (Thermo Scientific) through a Nanospray Flex Ion Source. Nanospray and S-lens voltages were set to 1.3–1.8 kV and 60 V, respectively. The capillary temperature was set to 250°C. Full scan MS survey spectra (*m*/*z* 360–1560) in profile mode were acquired in the Orbitrap with a resolution of 120 000 with a target value at 3 × 10^5^ and a maximum injection time of 50 ms. The 20 most intense peptide ions were fragmented in the higher-energy collisional dissociation (HCD) cell and analysed in the linear ion trap with a target value at 2 × 10^4^, a maximum injection time of 50 ms and a normalized collision energy at 29. Target ions selected for fragmentation were dynamically excluded for 25 s after two MS/MS events.

The protein database searches were launched using Proteome Discoverer^TM^ software from Thermo Fisher Scientific (v.2.4) and were performed with Mascot 2.6.2 (Matrix Science) against the mouse UniProt protein database (version 23 September 2019). The mass tolerances for precursor and fragment ions were set to 10 ppm and 0.05 Dalton, respectively. Trypsin was used as the enzyme allowing for up to one missed cleavage. Cysteine carbamidomethylation was specified as a fixed modification and methionine oxidation as a variable modification. Scaffold 5.2.2 was used to validate MS/MS-based peptide and protein identifications. Protein identifications were accepted if they could be established at greater than 95.0% probability and contained at least one identified peptide by the Protein Prophet algorithm [82]. Proteins that contained similar peptides and could not be differentiated based on MS/MS analysis alone were grouped to satisfy the principles of parsimony. Proteins sharing significant peptide evidence were grouped into clusters. The mass spectrometry proteomics data have been deposited to the ProteomeXchange Consortium via the PRIDE [83] partner repository with the dataset identifier PXD041687 and 10.6019/PXD041687. The data were validated by IP, where the interactions of SLMAP3 with lamin B1 and STRN, both detected in IP–MS, were confirmed.

### 5.7. Immunofluorescence and image processing

All images were acquired at the Cell Biology and Image Acquisition Core Facility at the University of Ottawa. For the slides with H&E staining and cell plate phase contrast, a Thermo Fisher EVOS FL Auto 2 was used. For fluorescence, we used the widefield Zeiss AxioObserver Z1 microscope, with the exception of images for Lamin B1 staining for which we used the widefield Zeiss AxioObserver D1 microscope. The images acquired were exported from Zen Blue and analysed in Fiji for image cropping. Background subtraction and contrast enhancement were applied for brightfield images. For the quantification of perinuclear staining, multiple images of each biological sample were analysed on CellProfiler by pipelines that we created and validated. In the pipelines, objects were created using 4′,6-diamidino-2-phenylindole (DAPI) staining. Further masking with MyoG or MHC staining selected cells positive for these markers. Expansion and shrinkage of DAPI objects followed by subtraction of the smaller object was used to generate three concentric bands of 10 pixels (pixel size 0.102 × 0.102 µm^2^) each. The mean intensity of the staining was measured in these bands. For pericentrin, AKAP6 and Kif5b, the bands were the NE, PE and cytoplasm (Cyto) objects. For Golgin-97 intensity measurement, these bands were shifted five pixels towards the cytoplasm, creating the PE, cytoplasm 1 (Cyto1) and cytoplasm 2 (Cyto2) objects. For Nesprin-1, lamin A/C and lamin B1, the mean intensity was measured on the NE object and in the DAPI object subtracted the NE, which was called the nucleoplasm object. The size of the NE object for lamin A/C and lamin B1 was four pixels, whereas for Nesprin-1, it was 10 pixels. For quantification of microtubule growth from the NE, we measured the mean intensity of α-tubulin staining in the NE object. For the fusion index, we used CellProfiler. For that, MHC staining was used to mask the nuclei. Myotubes containing at least two nuclei were considered and divided by the total nuclei identified in the field and multiplied by 100. Images with microtubule staining were acquired in Z-stack, and image deconvolution was applied using the Regularized Inverse Filter on Zen Blue. All other images were acquired in a single plane and followed image processing. The list of the antibodies used for immunofluorescence (IF) is as follows:

**Table:** 

primary antibodies				
**target**	**company**	**catalogue**	**host**	**dilution IF**
SLMAP	Novus Biologicals	NBP1-81397	rabbit	1:200
MyoG (F5D)	Santa Cruz	SC-12732	mouse	1:100
MHC	Developmental Studies Hybridoma Bank	MF20	mouse	1:50
GM130	BD Biosciences	610822	mouse	1:200
golgin 97	Proteintech	12640-1-AP	rabbit	1:100
calnexin	Novus Biologicals	NB100-1974	rabbit	1:200
lamin B1	Abcam	ab16048	rabbit	1:200
pericentrin	Covance/Biolegend	PRB-432C	rabbit	1:100
Akap9	Novus Biologicals	NBP1-89167	rabbit	1:150
α-tubulin (DM1A)	Millipore Sigma	T6199	mouse	1:300
Akap6	Millipore Sigma	HPA048741	rabbit	1:100
Kif5B [EPR10276(B)]	Abcam	ab167429	rabbit	1:100
nesprin-1	Wolfson Centre for Inherited Neuromuscular Disease	MANNES1A	mouse	1:100
lamin A/C	Wolfson Centre for Inherited Neuromuscular Disease	MANLAC3	mouse	1:4

**Table:** 

secondary antibodies				
**target**	**company**	**catalogue**	**host**	**dilution IF**
anti-mouse IgG (H + L) cross-adsorbed secondary antibody, Alexa Fluor 647	Invitrogen	A-21235	goat	1:300
anti-rabbit IgG (H + L) cross-adsorbed secondary antibody, Alexa Fluor 555	Invitrogen	A-21428	goat	1:300
anti-rabbit IgG H&L, Alexa Fluor 488	Abcam	ab150077	goat	1:200

### 5.8. Histology

For H&E staining, tissues were fixed on formalin 10%. The embedding, paraffinization, sectioning and staining were performed by the Louise Pelletier Histology Core Facility of the University of Ottawa.

### 5.9. Plasmids, cloning and viruses

The SLMAP3 mouse sequence was cloned as follows: cDNA from a mouse heart was used for SLMAP3 PCR amplification with Phusion^®^ High-Fidelity DNA Polymerase (BioLabs, cat. M0530) and using, respectively, the SLPN-forward and SLPN-reverse primers 5′-GATGCCAGCTTCTAGAGGGAGGACG and 5′-GGAATTCGATGCCGTCAGCCTTGGC. The SLPN-F contains an EcoRI restriction site before the start codon of SLMAP, and the SLP-R has an Xba I site after the stop codon. These two sites were used to clone SLMAP3 into the pcDNA3-CMV- (Invitrogen, cat. V79020) vector where we had previously cloned GFP between KpI and Eco RI restriction sites. The resulting GFP-SLMAP3, with the GFP in the N-terminus of SLMAP3, was sequenced by the DNA Sequencing Facility at the Ottawa Hospital, and the construct was validated by western blot and fluorescence analysis of transfected cells.

The GFP-SLMAP3 adenovirus was produced with the AdEasy Adenoviral Vector System from Agilent Technologies (cat. 240009), following the manufacturer protocol [84]. Briefly, the GFP-SLMAP3 from pCDNA3 was amplified with the primers GFP-shuttle 5′-GCGTCTAGAATGGACAAA GGAGAAGAACTC and pcDNA3.1-R 5′-CAACAGATGGCTGGCAACTAG and cloned into the pShuttle vector. The resulting plasmid was linearized with Pme I and transformed into BJ5183-AD cells, which were pre-transformed with the pAdEasy-1 vector. The resulting plasmid from the recombination of both vectors was digested with Pac I, followed by transfection in AD-293 packing cells. The virus production and infection were also performed according to the manufacturer’s guidelines [84].

The shRNAs for STRN3 were designed with the following target sequences: shRNA#1—CACTGGTAGTGCGGTAATTTA and shRNA#2—AGCAAGGCAGACAGCTATTAA. The SC control was designed with the target sequence AGGATAAGCGTCAACGAATAGGTGA. ShRNAs#1 and #2 target sequences were cloned into pLV[shRNA]-Neo-U6 vector, and the shRNA SC target sequence into pLV[shRNA]-Puro-U6 plasmid, all performed by VectorBuilder. The lentiviruses were packed in Lenti-X 293 T cells with the psPAX2 and pMD2.G plasmids following the Addgene protocol [85]. The transduction of C2C12 was performed following the Addgene protocol [86]. After 3 days, cells transduced with shRNA#1 and #2 lentiviruses were selected with G418 750 µg ml^−1^ for 6 days. Cells transduced with shRNA SC lentivirus were selected with puromycin 2.5 µg ml^−1^ for 3 days. The depletion of STRN3 and the shRNA control were validated by western blot.

### 5.10. Cell line generation

The polyclonal C2C12 lines with *slmap* genetic disruptions were generated, as described elsewhere [87], at the Genomic Editing and Molecular Biology Core Facility in the Faculty of Medicine at the University of Ottawa. The following guide RNAs targeting exon 3 were used for the generation of CRISPR/Cas9 knockout: 5′-AGTCGGGCTTCCATACCATC and 5′- ATACTCACTCTGAACGAAGT. These guides were cloned into pLentiCRISPRv2 (Addgene plasmid 52961 [87]), and the transduced cells were selected with puromycin 2.5 µg ml^−1^ for 3 days. The gene cleavage was confirmed with T7 endonuclease I (NEB) using the following primer pairs: 5′-CGGCCAGGAGAGACTATCAC and 5′-TCCAAGTCTCATGGCGTGTG. We isolated monoclonal colonies from the cell pools generated with both guide RNAs by limiting diluting according to Addgene [88]. We confirmed the selection of knockout colonies by western blot and immunofluorescence. For non-targeting control (NTC), we used the pLentiCRISPRv2 carrying a guide RNA targeting GFP (Addgene plasmid 86153 [89]), and the transduced C2C12 cells were selected with puromycin. No statistically significant differences were identified between not transduced and NTC C2C12 WT myoblasts in terms of differentiation, myotube formation and MTOC switch to the NE after differentiation.

### 5.11. Statistical analysis

All the statistical analyses were performed on GraphPad Prism. Data were represented by mean and standard error of mean. For analyses with one variable with two groups, we used *t*‐test. For three or more groups, we used one-way ANOVA followed by Newman–Keuls post-test. Tests with multiple variables were performed with two-way ANOVA followed by Bonferroni post-test. Statistical significance is indicated as *p < 0.05, **p < 0.01, ***p < 0.001 and n.s. for not significant. At least three biological samples were used for all statistical analyses in this study.

## Data Availability

The RNA-seq from this study have been deposited in NCBI’s Gene Expression Omnibus and are accessible through GEO Series accession number GSE230748 (https://www.ncbi.nlm.nih.gov/geo/query/acc.cgi?acc=GSE230748). The IP–MS results have been deposited to the ProteomeXchange Consortium via the PRIDE partner repository with the dataset identifier PXD041687 and 10.6019/PXD041687. The raw images of all western blots of this study are available in electronic supplementary material, S2. Supplementary material is available online [90].
